# Diving response after a one-week diet and overnight fasting

**DOI:** 10.1186/s12970-016-0134-y

**Published:** 2016-05-31

**Authors:** Giovanna Ghiani, Elisabetta Marongiu, Sergio Olla, Marco Pinna, Matteo Pusceddu, Girolamo Palazzolo, Irene Sanna, Silvana Roberto, Antonio Crisafulli, Filippo Tocco

**Affiliations:** Department of Medical Sciences, School of Sport Medicine, Sport Physiology Lab, University of Cagliari, Via Porcell 4, 09124 Cagliari, Italy

**Keywords:** Cardiac output, Free-diving, Fasting, Breakfast

## Abstract

**Background:**

We hypothesized that overnight fasting after a short dietary period, especially with carbohydrates, could allow performing breath-hold diving with no restraint for diaphragm excursion and blood shift and without any increase of metabolism, and in turn improve the diving response.

**Methods:**

During two separate sessions, 8 divers carried out two trials: (A) a 30-m depth dive, three hours after a normal breakfast and (B) a dive to the same depth, but after following a diet and fasting overnight. Each test consisted of 3 apnea phases: descent, static and ascent whose durations were measured by a standard chronometer. An impedance cardiograph, housed in an underwater torch, provided data on trans-thoracic fluid index (TFI), stroke volume (SV), heart rate (HR) and cardiac output (CO). Mean blood pressure (MBP), arterial O_2_ saturation (SaO_2_), blood glucose (Glu) and blood lactate (BLa) were also collected.

**Results:**

In condition B, duration of the static phase of the dive was longer than A (37.8 ± 7.4 vs. 27.3 ± 8.4 s respectively, *P* < 0.05). In static phases, mean ∆ SV value (difference between basal and nadir values) during fasting was lower than breakfast one (−2.6 ± 5.1 vs. 5.7 ± 7.6 ml, *P* < 0.05). As a consequence, since mean ∆ HR values were equally decreased in both metabolic conditions, mean ∆ CO value during static after fasting was lower than the same phase after breakfast (−0.4 ± 0.5 vs. 0.4 ± 0.5 L · min^−1^ respectively, *P* < 0.05). At emersion, despite the greater duration of dives during fasting, SaO_2_ was higher than A (92.0 ± 2.7 vs. 89.4 ± 2.9 % respectively, *P* < 0.05) and BLa was lower in the same comparison (4.2 ± 0.7 vs. 5.3 ± 1.1 mmol∙L^−1^, *P* < 0.05).

**Conclusions:**

An adequate balance between metabolic and splancnic status may improve the diving response during a dive at a depth of 30 m, in safe conditions for the athlete’s health.

## Background

To obtain lengthy apnoeas divers try to minimise oxygen consumption and to this end nutritional status may also affect their results. Generally, a pre-competition or training meal eaten 1–4 h before exercise should provide adequate fluids and nutrients to ensure optimal carbohydrate availability and hydrated status without gastrointestinal distress [1, 2]. However, splancnic filling may contrast diaphragmatic excursion and pulmonary expansion. As a result, it is likely that many elite divers prefer to fast overnight before diving. Moreover, from a metabolic point of view, fasting may increase oxygen consumption as lipid stores are metabolized and reduce the relative amount of carbon dioxide (CO_2_) produced. As a consequence, CO_2_ pressure will be lowered and this may delay the involuntary diaphragmatic contractions with benefit for divers. However, on the reverse side, the termination of apnoea at maximal breakpoint will occur at lower end-tidal oxygen pressures with an increased risk of black-out [3]. The authors of the afore-mentioned study found this risk during breath-holds performed after prolonged exercise, but when fasting was tested prior to performing static apnoeas their viewpoints varied. While Lindholm et al. [4] measured lower O_2_ levels at breakpoint during fasting than after ingestion of carbohydrates, suggesting an increased risk of syncope, Schagatay and Lodin-Sundström [5] found that fasting might improve static apnoea performance without any risk of syncope, because the oxygen levels measured at the end of apnoeas after fasting were not lower than after eating and in any case not low enough to cause a risk of black-out.

On the basis of the above, during breath-hold diving in the sea the risk of an inadequate balance between metabolic and splancnic conditions may also be higher because of augmented oxygen consumption and the presence of “blood shift”. In fact, the first may increase the risk of syncope and the second may be hampered by splancnic filling. We hypothesized that overnight fasting after a short dietary period, especially with carbohydrates, could allow performing breath-hold diving with no restraint for diaphragm excursion and blood shift and without any increase of metabolism, and in turn improve the diving response. For this reason, we measured hemodynamic and metabolic parameters in breath-hold divers who performed free-diving in the sea in two conditions: after overnight fasting and after breakfast.

## Methods

### Experimental design

Two trials were carried out, in different days and at the same time of the day, by each diver: (A) a dive at 30 m-depth three hours after breakfast and (B) a dive at the same depth but after following a specific diet and overnight fasting. Each trial was accomplished according to this procedure: After 5 min of immersion at the surface performing normal breathing by means of a snorkel (baseline level), divers performed a descent to 30 m depth followed by variable durations of static apnoea (SA), depending on individual breath-hold skills, subsequent ascent and emergence from the water. In order to study the hemodynamic changes during the two different conditions, static and dynamic apnoea, divers were asked to perform a maximal SA at the bottom.

Ballast was used during dives and its weight was chosen based on the subjects’ personal experience. Divers were instructed to follow a guiding rope to maintain the correct direction during dives. Descent and ascent were performed at a speed of about 1 m∙s^−1^ by kicking with bi-fins through all the depths of dives. The mean speed of descent and ascent was calculated by dividing the covered distance by duration of descent and ascent which was recorded using a standard waterproof chronometer. All divers were asked to begin immersion after maximal inspiration without performing neither hyperventilation or packing manoeuvres.

### Subjects

Eight healthy male well-trained divers (see Table 1 for their anthropometric and metabolic parameters) were recruited. Information on the study procedures was provided to all participants and the study was carried out according to the Declaration of Helsinki. Written informed consent was obtained before subjects entered the study.

**Table 1:** Anthropometric, nutritional and metabolic parameters of the divers involved in the study

Divers	Age (years)	BM (kg)	Height (cm)	FFM (kg)	FM (kg)	TBW (%)	PAL	Breakfast (kcal)	TSH (mU/L)	FT_3_ (pg/mL)	FT_4_ (pg/mL)	VO_2_max (mL/kg/min)
1	47	74	176.0	63.7	10.3	63.0	1.7	600	1.7	4.01	12.9	45.2
2	45	75.7	179.5	60.3	15.4	58.7	1.6	500	1.8	3.89	13.4	48.5
3	44	64.9	170.5	55.9	9.0	63.0	1.7	600	2.1	3.86	14.9	52.2
4	39	68.0	171.5	59.9	8.1	64.4	1.6	400	1.0	4.02	14.1	47.1
5	41	60.0	166.0	51.1	8.9	62.2	1.7	500	3.0	4.68	15.1	44.3
6	42	53.5	163.0	47.4	6.1	64.7	1.5	400	1.3	4.56	12.7	39.2
7	44	49.9	160.0	39.0	10.9	56.9	1.5	400	2.4	3.97	13.3	46.7
8	51	58.7	165.0	50.5	8.3	62.9	1.5	400	1.8	3.99	13.7	44.3
Mean ± SD	44.1 ± 3.7	63.1 ± 9.2	168.9 ± 6.6	53.5 ± 8.1	9.6 ± 2.7	62.0 ± 2.7	1.62 ± 0.1	475 ± 88.6	1.89 ± 0.6	4.12 ± 0.3	13.8 ± 0.9	45.9 ± 3.8

*BM* body mass, *FFM* free fat mass, *FM* fat mass, *TBW* total body water, *PAL* physical activity level, *TSH* thyroid stimulating hormone, *FT*_*3*_ free triiodothyronine, *FT*_*4*_ free thyroxine, *VO*_*2*_ *max* maximum oxygen uptake

### Food frequency questionnaire

Subjects were asked to fill in a semi-quantitative food-frequency questionnaire [6] to assess their usual intake, both in calories and nutrients. The questionnaire consisted of 16 printed forms and 16 pages with coloured photos of the most common foods and dishes in the Italian diet. Instructions are included.

### Calorie needs

An itemized interview about divers’ lifestyle was conducted and a Physical Activity Level (PAL) was established to calculate the food requirement for each of them.

### Anthropometric measurements

For each subject basic anthropometric measurements including body mass (BM, kg) fat mass (FM, kg), fat-free mass (FFM, kg) were taken. Body composition was assessed by means of the plicometry method (triceps, biceps, sub scapular, and supra iliac skin folds) and BM was measured by a scale (Seca 709, Hamburg, Germany). The skin fold thickness was measured with a Lange skin fold calliper (Beta technology, Mission St., Santa Cruz, USA) and FM % was predicted by using the Durnin and Womersley [7] skin fold thickness equation. Skin fold thickness was measured on the right side of the body by the same experienced technician. Measurements were always taken at the same time of the day and the participants were instructed to abstain from exercise and to avoid alcohol 24 h prior to the test. In addition, participants were asked not to drink any fluid 4 h prior to their test and urinate within 30 min of test. Skin fold thickness was taken three times and the average value was used in the equation. To check the hydration status of divers we also estimated the total body water (TBW) by the use of the following formula: TBW = 0.737 × FFM [8]. Also, basic parameters as thyroid status and maximal oxygen uptake (VO_2_max) were collected to check the metabolic homogeneity of the sample. Free FT3, FT4 and TSH levels were measured by automatic ultrasensitive chemiluminescence assays (Ortho Clinical Diagnostic Sp, Milan, Italy). Normal values: FT3: 3.2–6.1 pg/mL; FT4: 7.7–21.9 pg/mL; TSH: 0.2–3.0 mU/L. Each subject underwent a preliminary incremental treadmill running test (RunRace, Tecnogym, Forlì, Italy) to measure VO_2_max. After 3 min of rest in the standing position, the subject started to run at 6 km h^−1^ and speed was increased by 1 km · h^−1^ every 3 min, until exhaustion. Achievement of VO_2_max was considered as the attainment of at least two of the following criteria: 1) a plateau in VO_2_, despite increasing speed; 2) a respiratory exchange ratio >1.10; and 3) a heart rate ± 10 beats · min-1 of predicted maximum heart rate (HR) calculated as 220-age [9].

### Dietary protocol

A personalized isocaloric diet providing all the essential energy and nutrients required was given to each subject participating in the study, with the advice to follow it for one week before test B. The diet provided a personalized number of calories amounting to about 20 proteins, 50 carbohydrates and 30 % fats. Total daily intake was divided into three main meals: breakfast (30), lunch (45) and dinner (25 %). Each participant was also given a particular breakfast to have on the day of the test A. This particular breakfast gave them the 20 % of their daily calorie needs and it comprised a combination of complex and simple carbohydrates, for example: a cup of tea with brown bread, fruit and jam or honey.

### Assessment of metabolic and hemodynamic variables

Hemodynamic and metabolic changes were assessed during several meetings which took place on the coast near Cagliari, in the Mediterranean Sea. During all sessions, surface and 30 m-depth water temperatures ranged between 25 and 22 °C. Before immersion, fingertip arterial oxygen saturation (SaO_2_) (Biox 3740 Pulse Oximeter, Ohmeda, USA), arterial blood pressure (standard manual sphygmomanometer), blood lactate (BLa, Lactate pro, Arkray Inc., Kyoto, Japan) and blood glucose concentrations (Glu, FreeStyle Optium Glucose Meter, Abbott Diabetes Care Inc., Alameda, USA) were measured. At the end of the dives, divers sat on the edge of the boat and a single measurement of blood pressure was collected by a standard sphygmomanometer (performed in <20 s). Further, SaO_2_ values (in <10 s) were detected by means of the oximeter set with a 3-s averaging function, and the first value which appeared on the display was considered. Blood pressure and SaO_2_ measurements were taken in the shortest possible time in an attempt to represent the apnoea effects at the end of dives. Then, blood samples were obtained with a finger prick and Glu and BLa values were respectively gathered within 30 and 180 s of emergence from the water. The impedance cardiography (IC) measurements were provided by a miniaturized device (New Core, 2C Technologies Inc., Cagliari, Italy) connected to the subjects’ neck and chest by 8 electrodes which were waterproofed using surgical 15x10 cm patches (Plastod, Bologna, Italy). Then, subjects wore a diving wetsuit (5 mm of thickness), a diving mask and a snorkel. To apply the IC during underwater apnoea, the New Core was placed in an underwater torch, which was waterproof to a depth of 90 m. The efficacy of the New Core had been previously tested on 18 male subjects and validation details can be found in a recent work by Tocco et al. [10]. IC has been commonly employed in resting and exercising subjects [11, 12]. Thus, the impedance method provides reliable data on thoracic fluid index (TFI), left ventricular ejection time (VET), SV, HR and CO. The SV/VET ratio was also assessed and considered as an index of myocardial performance [13]. Systemic vascular resistance (SVR) was obtained by dividing MBP (calculated as diastolic blood pressure + 1/3systolic blood pressure-diastolic blood pressure) by CO.

### Data analysis

Impedance and ECG recorded traces were analyzed employing a digital chart recorder (ADInstruments, PowerLab 8sp, Castle Hill, Australia). We previously set up an impedance traces recording and processing method which is described in detail in previous works [14, 15]. Beat-to-beat hemodynamic collected data were averaged for each phase (rest, descent, static and ascent). To curtail the inter-individual variance and to highlight small perturbations in parameters, the changes with respect to the rest value were calculated and expressed as delta (Δ). Results are given as mean ± SD. Paired analysis on the metabolic and hemodynamic data collected at rest and at emersion was used to compare breakfast and fasting conditions. Repeated measures two-way ANOVA was applied to find significant differences among the three phases of each dive during the two conditions A and B. Newman-Keuls post-hoc was performed when appropriate. Statistics were performed using commercially available software (Graph-Pad Prism Software, San Diego CA USA). Significance was set at α value < 0.05.

## Results

The protocol was completed by all divers and no symptoms or blackouts during or following dives were reported. Divers anthropometric values, metabolic and nutritional parameters are shown in Table 1. Absolute values of metabolic and hemodynamic data during rest preceding dives are reported in Table 2.

**Table 2:** Absolute hemodynamic and metabolic data in divers at rest preceding the dives

Parameter (Units)	BLa (mmol∙L^−1^)	SaO_2_ (%)	Glu (mg · dL^−1^)	HR (bpm)	SV (ml)	CO (L∙min^−1^)	SV/VET (ml · sec-^1^)	MBP (mmHg)	SVR (dyne · s/cm^5^)	TFI (Ohms)
Breakfast	1.3 ± 0.6	98.9 ± 0.4	81.1 ± 11.8	67.2 ± 6.0	72.0 ± 9.8	4.6 ± 0.5	234.2 ± 47.9	90.8 ± 10.2	1586.6 ± 305.9	31.5 ± 2.0
Fasting	1.5 ± 0.6	98.6 ± 0.5	86.8 ± 7.8	66.3 ± 4.0	74.8 ± 8.3	5.0 ± 0.6	265.9 ± 25.3	89.2 ± 7.9	1497.4 ± 289.4	30.3 ± 1.4

Values are mean ± SD; *BLa* blood lactate, *SaO*_*2*_ arterial oxygen saturation, *Glu* blood glucose, *HR* heart rate, *SV* stroke volume, *CO* cardiac output, *SV/VET* stroke volume/ventricular ejection time, *MBP* mean blood pressure, *SVR* systemic vascular resistance, *TFI* thoracic fluid index

**Fig. 1:**
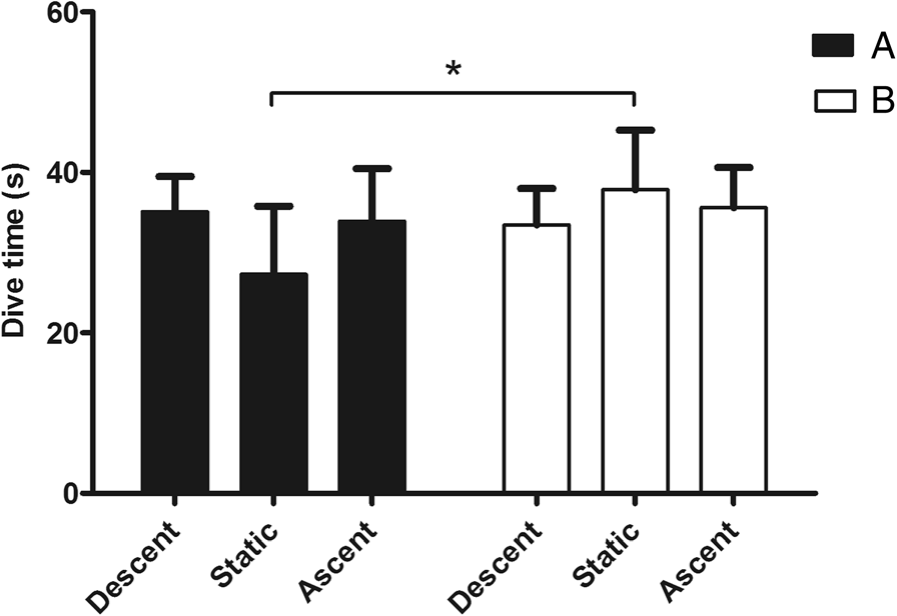
Comparison between durations of the 3 phases (descent, static and ascent) of the protocol after breakfast (**a**) and fasting (**b**). Values are mean ± SD. Asterisk (*) indicates *P* < 0.05 vs. corresponding time point of static

**Fig. 2:**
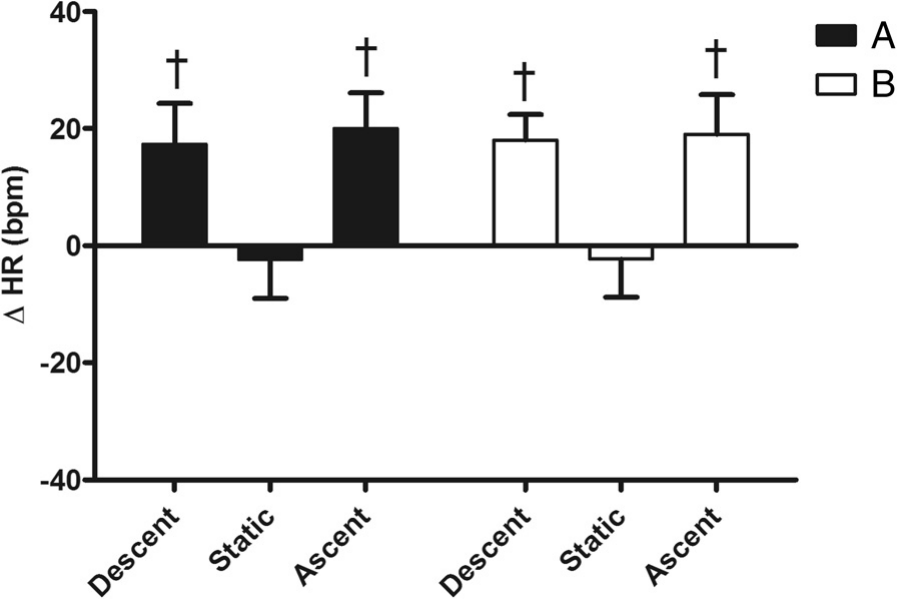
Changes from rest in heart rate (ΔHR) during the 3 phases (descent, static and ascent) of the protocol after breakfast (**a**) and fasting (**b**). Values are mean ± SD. Dagger (†) indicates *P* < 0.05 vs. static in the same condition

**Fig. 3:**
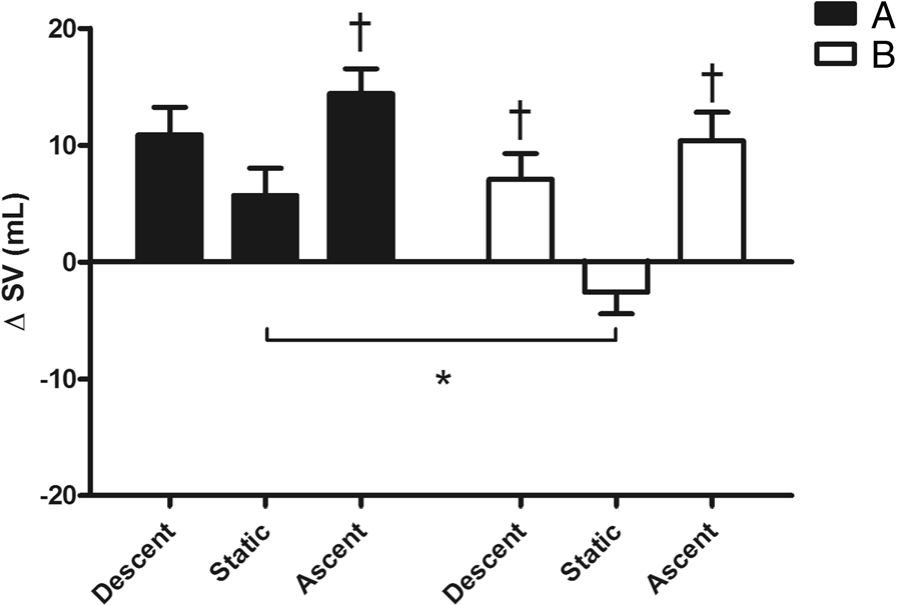
Changes from rest in stroke volume (ΔSV) during the 3 phases (descent, static and ascent) of the protocol after breakfast (**a**) and fasting (**b**). Values are mean ± SD. Asterisk (*) indicates *P* < 0.05 vs. corresponding time point of static. Dagger (†) indicates *P* < 0.05 vs. static in the same condition

**Fig. 4:**
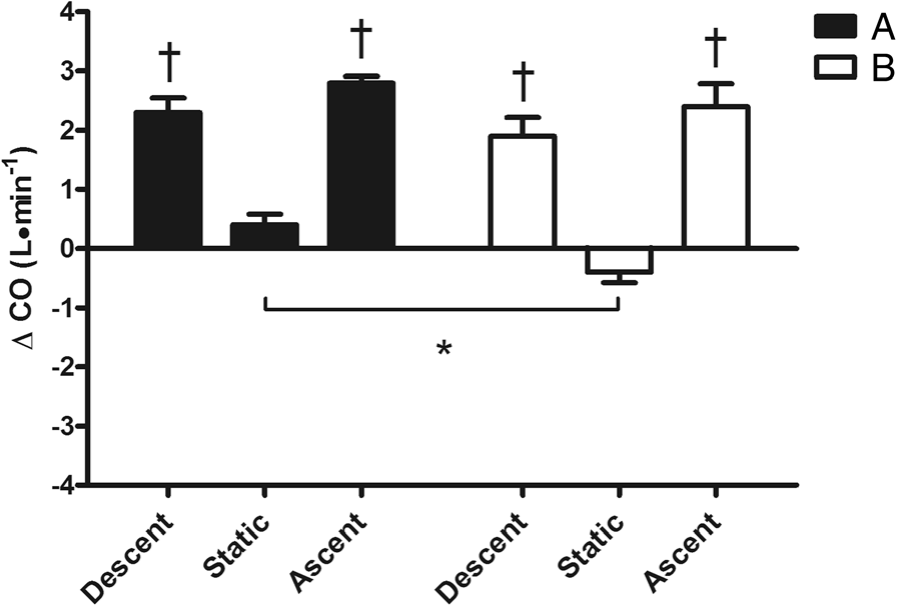
Changes from rest in cardiac output (ΔCO) during the 3 phases (descent, static and ascent) of the protocol after breakfast (**a**) and fasting (**b**). Values are mean ± SD. Asterisk (*) indicates *P* < 0.05 vs. corresponding time point of static. Dagger (†) indicates *P* < 0.05 vs. static in the same condition

**Fig. 5:**
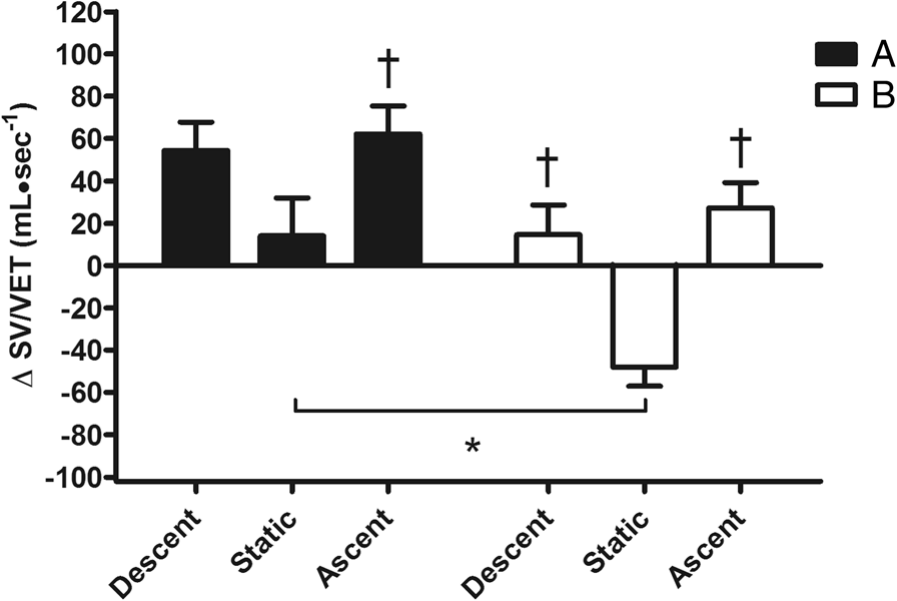
Changes from rest in stroke volume/ventricular ejection time ratio (ΔSV/VET) during the 3 phases (descent, static and ascent) of the protocol after breakfast (**a**) and fasting (**b**). Values are mean ± SD. Asterisk (*) indicates *P* < 0.05 vs. corresponding time point of static. Dagger (†) indicates *P* < 0.05 vs. static in the same condition

**Fig. 6:**
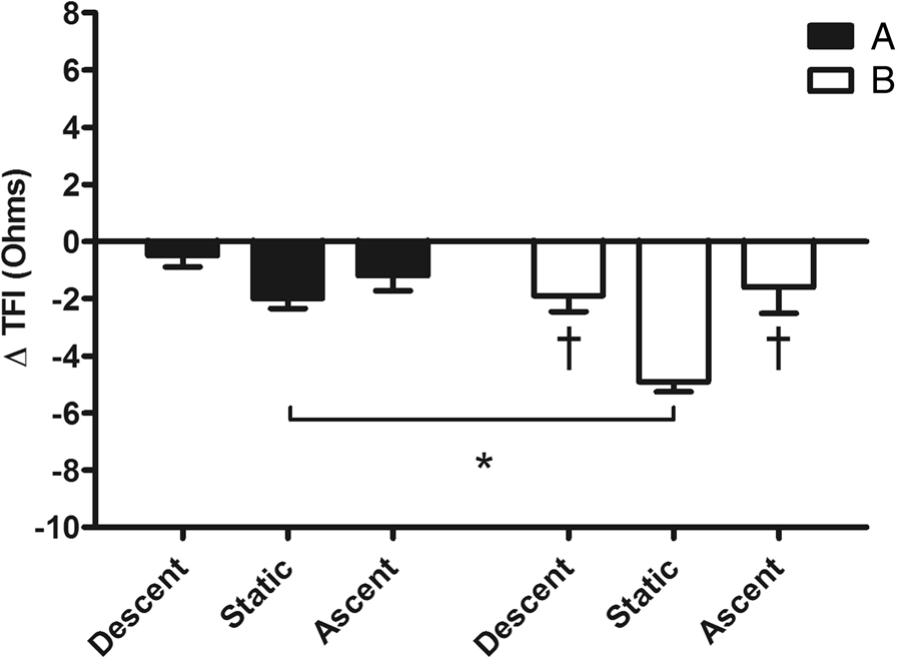
Changes from rest in thoracic fluid index (ΔTFI) during the 3 phases (descent, static and ascent) of the protocol after breakfast (**a**) and fasting (**b**). Values are mean ± SD. Asterisk (*) indicates *P* < 0.05 vs. corresponding time point of static. Dagger (†) indicates *P* < 0.05 vs. static in the same condition

Figures 1, 2, 3, 4, 5 and 6 show the comparison among the variables measured during the 3 phases (descent, static and ascent) of dives performed in the two experimental conditions A and B.

Figure 1 shows in detail that during B condition, the duration of the static phase of dives was greater than A (37.8 ± 7.4 vs. 27.3 ± 8.4 s respectively, *P* < 0.05). Nevertheless, no statistical difference in chronotropism between the two static phases was detected (Fig. 2). However, in both conditions A and B, HR levels at static were largely lower than at descent and ascent. During static phases SV was lower after fasting with respect to breakfast (−2.6 ± 5.1 vs. 5.7 ± 7.6 ml, *P* < 0.05) while in both A and B conditions SV was significantly augmented in both descent and ascent compared to static (Fig. 3). Thus, as a consequence in both chronotropic and inotropic responses, the CO values during static after fasting were lower than the same phase after breakfast (−0.4 ± 0.5 vs. 0.4 ± 0.5 L · min^−1^ respectively, *P* < 0.05). Nevertheless, CO was reduced during static in both A and B in comparison to descent and ascent phases (Fig. 4). Furthermore, a significant difference between breakfast and fasting conditions was seen in the SV/VET rate during static (14.1 ± 50.8 vs. -48.1 ± 25.6 mL · sec^−1^ respectively, *P* < 0.05), i.e. this index of myocardial performance was impaired during static after fasting (Fig. 5). On the other hand, SV/VET was augmented during descent and ascent phases with respect to static in both conditions (Fig. 5). TFI, an index of venous return and, in this case, an index of blood shift, was reduced (i.e. venous return increased) throughout all phases of the dive but during the static phase after fasting TFI decreased more than breakfast (−4.9 ± 1.0 vs. -2.0 ± 1.0 Ω respectively, *P* < 0.05, Fig. 6).

Table 3 reports data of all parameters gathered at emersion. As expected, considering data from Fig. 1, the mean duration time (MDT) of 30 m-depth dives was longer for B than A (103.8 ± 13.8 vs. 93.4 ± 12.1 s respectively, *P* < 0.05). Interestingly, despite the longer duration of dives during fasting, SaO_2_ was higher after B than A (92.0 ± 2.7 vs. 89.4 ± 2.9 % respectively, *P* < 0.05) and BLa was lower in the same comparison (4.2 ± 0.7 vs. 5.3 ± 1.1 mMol∙L^−1^ respectively, *P* < 0.05). Finally, Glu, MBP and SVR values were not differently affected by treatments A and B.

**Table 3:** Data gathered at emersion

Parameter (Units)	MDT (sec)	BLa (mmol∙L^−1^)	SaO_2_ (%)	Glu (mg · dL^−1^)	MBP (mmHg)	SVR (dyne · s · cm^−5^)
Breakfast	93.4 ± 12.1	5.3 ± 1.1	89.4 ± 2.9	97.3 ± 14.4	115.2 ± 7.9	1225.9 ± 145.6
Fasting	103.8 ± 13.8*	4.2 ± 0.7*	92.0 ± 2.7*	100.3 ± 17.1	114.6 ± 6.7	1276.6 ± 194.7

Values are mean ± SD; *MDT* mean dives time, *BLa* blood lactate, *SaO*_*2*_ arterial oxygen saturation, *Glu* blood glucose, *MBP* mean blood pressure, *SVR* systemic vascular resistance. * = *p* < 0.05

## Discussion

This research highlights the importance of nutrition management during free-diving apnoea in the sea, considering the metabolic and hemodynamic implications. In previous research on this topic, dietary impact on diving response was investigated in laboratory settings only, either in static [4, 5] or dynamic apnoeas [3] and authors particularly investigated respiratory and metabolic parameters. The main result of this study was that an adequate balance between metabolic and splancnic status (overnight fasting after a short dietary period, test B) improved the diving response during a dive at 30 m-depth with a prolonged apnoea static phase at the bottom. It is noteworthy that after emersion from test B, SaO_2_ and BLa values were respectively higher and lower than after test A (i.e. after breakfast) and divers showed normal glucose and blood pressure levels (Table 3). Thus this ameliorated performance was safely obtained by divers.

From the analysis of the hemodynamic changes assessed by means of impedance cardiography during dives (graphs from 2 to 6) it can be noted that a typical diving response with bradycardia, CO decrease and blood shift (i.e. TFI decreased) occurred during the static phase of test B only. Indeed, during the same phase of test A, despite the little HR reduction, CO was slightly increased because of SV increment, thus the oxygen-sparing effect was blunted. In fact, as underlined above, at emersion during breakfast condition divers showed lower SaO_2_ and higher BLa values than test B. In our opinion, the poor diving response of test A probably occurred as a result of improved myocardial contractility as revealed by the SV/VET increase. In this regard, it is important to note that an inotropic response was particularly evident during the descent and ascent phases of both conditions A and B, in which SV/VET, HR, SV and CO Δ values were significantly higher compared to rest and static phases. As recently reported by Marongiu et al. [16] in a similar experiment, this hemodynamic scenario was probably caused by the combined effect of the vagal withdrawal and sympathetic activation linked to the exercise during ascent and descent phases.

In contrast to the aforementioned study, in this present research, during the static phase in fasting condition, the diving response was well evoked with a CO decrease due to a simultaneous HR and SV decrement. In our opinion, the fasting condition determined an increased availability of circulatory resources compared to the breakfast status. This outcome is in agreement with results by Lemaitre et al. [17] who reported that the diving response was strong enough to override the stimulus of muscular exercise in elite divers that showed bradycardia performing deep dives during a real free-diving in the sea. Unfortunately, the authors of this research did not report any information on the metabolic status of their divers. From this hemodynamic point of view, results of the present study suggest that fasting status is the optimal condition in which to perform short dives because it ensures optimal diving response without any decrease in plasma glucose levels. However, even though in test A breakfast was consumed three hours prior diving, this cannot completely rule out an amount of splanchnic blood pooling, potentially leading to discomfort and hence a shorter apnea time than B.

The outcome during prolonged apnoeas cannot be inferred from the present data and prior to this study, blood glucose concentrations had never been investigated during free-diving in humans. However, interesting data are available from research conducted on diving mammals. Plasma glucose, glucagon and insulin concentrations were measured by Robin et al. [18] in harbour seals before, during a 6-min dive and a 30 min recovery period in two experimental conditions: during fasting (12 h prior to dive) and following intravenous glucose. The authors of the above-quoted study reported that in harbour seals, during diving in fasting state, plasma glucose, glucagon and insulin were not different from pre-diving levels, while during post-diving plasma glucagon increased (without any insulin change), leading to an increase in glucose availability towards non-insulin tissues like the brain. Interestingly, in our study on humans, at emersion from 30 m depth in fasting condition divers showed higher Glu levels than those at rest preceding dives and they were not different from levels in breakfast condition. Diving mammals such as harbour and elephant seals often dive in a prolonged fasting state because dives are mainly performed for foraging [19], and in these mammals the glucoregulation seems to be an important adaptation to diving [18]. Thus, from a teleological point of view, also in human divers, as primarily supposed by Scholander [20] as regards bradycardia and vasoconstriction for determining an oxygen sparing effect, the maintaining of glucose homeostasis in fasting state may be an important adaptation integrated to the diving response. Moreover, unlike diving performances performed by humans for competitive purposes like static apnoea or no-limits immersions, breath-hold diving activities are normally matched to dynamic exercise and this leads to a circulating epinephrine increase which is known to be an inhibitor of insulin secretion and a stimulator of glucagon release. In this sense, overall immersions excessively close to the breakfast are not necessary and may instead cause a blunted diving response. Thus, overnight fasting may be an advantageous condition to perform short immersions in humans, similar to what usually occurs in diving mammals. Data suggest that the exercise performed during free-diving induced hormonal changes that involve glucoregulation and this seems to be an important adaptation to diving. Moreover, the data about thyroid function in harbour seals reported by Weingartner et al. [21] showed that also thyroid hormones play an important role in modulating the at-sea metabolism, as hyperthyroid seals studied by authors showed a more pronounced cardiovascular response because of an augmented oxygen consumption. In this sense, to exclude any metabolic dysfunction in our divers potentially able to influence the cardiovascular diving response, we also investigated divers’ basic parameters as thyroid status and VO_2_max. As shown in Table 1, all divers had normal level of thyroid hormones and a similar aerobic capacity.

### Limitation of the study

Undoubtedly several factors have to be considered as potential limits of the present study. The main ones regard the limited number of divers involved in the study and the short duration of dives. Thus further studies are needed to confirm our conclusions and in particular a larger sample of divers than ours and a protocol with more prolonged apnoeas in real free-diving conditions are recommended. Another limitation of the present investigation could be the accuracy of IC to assess CO in submersed environment taking into account the potential redistribution in extracellular and intracellular fluid due to the increasing depth. In this regard Ayme et al. [22] reported a hemodilution induced by the increase in hydrostatic pressure after 1-h cycling exercise in water compared to the same exercise in land. However it is reasonable to hypothesize that the short duration of our protocol would not have evoked a similar response. In another study [23] CO data available in underwater environment have been assessed by means of echocardiography even if under quite different experimental conditions to make a comparison. Certainly it would be useful in the future. Finally, to test the efficacy of the overnight fasting strategy, pre and post treatment measurements of the total lung capacity and/or forced vital capacity would be useful as well as measurements of the resting oxygen uptake in comparison between conditions A and B. Unfortunately we were not able to perform these measurements.

## Conclusions

Data from the present study underline how the metabolic and hemodynamic adjustments of the diving response is related to an adequate balance between metabolic and splancnic status, which may improve the diving response during a single dive at a depth of 30 m, in safe conditions for the athlete’s health. Albeit larger scale studies are required to confirm our results, they suggest the possibility that divers could safely improve their performance through a better management of their diet related to training and competition.

## Abbreviations

ANOVA, Analysis of variance; BLa, Blood lactate; BM, Body mass; CO, Cardiac output; CO_2_, Carbon dioxide; FFM, Fat-free mass; FM, Fat mass; FT_3_, Free triiodothyronine; FT_4_, Free thyroxine; Glu, Blood glucose; HR, Heart rate; IC, Impedance cardiography; MBP, Mean blood pressure; MDT, Mean duration time; PAL, Physical activity level; SA, Static apnoea; SaO_2_, Arterial O_2_ saturation; SV, Stroke volume; SVR, Systemic vascular resistance; TBW, Total body water; TFI, Trans-thoracic fluid index; TSH, Thyroid stimulating hormone; VET, Ventricular ejection time; Δ, Delta
